# Intraoperative Photodynamic Diagnosis Using Talaporfin Sodium Simultaneously Applied for Photodynamic Therapy against Malignant Glioma: A Prospective Clinical Study

**DOI:** 10.3389/fneur.2018.00024

**Published:** 2018-01-30

**Authors:** Kazuhide Shimizu, Masayuki Nitta, Takashi Komori, Takashi Maruyama, Takayuki Yasuda, Yu Fujii, Ken Masamune, Takakazu Kawamata, Taketoshi Maehara, Yoshihiro Muragaki

**Affiliations:** ^1^Department of Neurosurgery, Tokyo Medical and Dental University, Tokyo, Japan; ^2^Faculty of Advanced Techno-Surgery, Institute of Advanced Biomedical Engineering and Science, Tokyo Women’s Medical University, Tokyo, Japan; ^3^Department of Neurosurgery, Tokyo Women’s Medical University, Tokyo, Japan; ^4^Department of Laboratory Medicine and Pathology (Neuropathology), Tokyo Metropolitan Neurological Hospital, Tokyo, Japan; ^5^Department of Neurosurgery, Shinshu University School of Medicine, Matsumoto, Japan

**Keywords:** talaporfin sodium, NPe6, photodynamic diagnosis, photodynamic therapy, malignant glioma, glioblastoma

## Abstract

**Objective:**

The goal of this study was to demonstrate the feasibility of intraoperative photodynamic diagnosis (PDD) of malignant glioma using the fluorescence from talaporfin sodium (TS), which is used simultaneously for photodynamic therapy (PDT).

**Methods:**

Patients with suspected primary malignant glioma who were eligible for surgical removal of the tumor and PDT with TS were enrolled in this prospective study. Tissue samples were obtained from the contrast-enhanced (CE) region and from the surrounding non-contrast-enhanced (NCE) marginal tissue at the boundary between the tumor and normal tissue. The excised samples were set into a fluorescence measurement system, which consisted of a semiconductor laser with a 400-nm wavelength for excitation, and a compact spectrometer for detection, which were applied and received through a custom-made probe consisting of coaxial optical fibers. The fluorescence spectrum was obtained, and peak intensity was calculated. Tumor cellularity was histopathologically analyzed and semi-quantitatively classified into four (0–3) categories.

**Results:**

86 samples from 17 surgical cases were available for fluorescence measurement and analysis. The fluorescence from TS had a single peak at 664 nm that was easily distinguished from the 400-nm excitation light. Samples from the CE regions showed higher fluorescence intensity than those from the NCE regions (*P* < 0.001). DAPI staining and fluorescence microscopy confirmed that cells in the CE regions showed red fluorescence in their cytoplasm. The fluorescence was notably strong along vascular endothelium. CE samples from newly diagnosed versus recurrent cases showed no difference in fluorescence intensity (*P* = 0.26). Among all samples (CE and NCE combined), the fluorescence intensity was very high in those of histopathological class 3, and a trend of increased fluorescence according to histopathological class (*P* < 0.001) was shown. Differences between class 0 and 3 (*P* < 0.001), class 1 and 3 (*P* < 0.001), and class 2 and 3 (*P* = 0.018) were significant.

**Conclusion:**

Intraoperative simultaneous PDD and PDT with TS can be performed for patients with malignant glioma. The blue excitation light that is used for 5-aminolevulinic acid PDD can be used for our technique (TS-PDD). The strong fluorescence from pathologically malignant tissues may be due at least in part to the involvement of microvascular structures.

## Introduction

Malignant glioma is a brain tumor characterized by its infiltrative nature. The most common type of malignant glioma is glioblastoma multiforme (GBM), which has a grim prognosis even after intensive surgical treatment and adjuvant radiation and chemotherapies. Among these therapies, aggressive surgical removal is associated with the best prognosis for GBM (1–5). Nevertheless, GBM recurs locally in 50–80% of the cases, and this recurrence is the leading cause of tumor progression (6, 7). Thus, the preferred treatment for this disease is maximal surgical cytoreduction while preserving the adjacent anatomical structures of the brain, followed by local treatment.

Photodynamic techniques including intraoperative photodynamic diagnosis (PDD) and photodynamic therapy (PDT) for malignant gliomas are under extensive clinical investigation. In particular, the use of 5-aminolevulinic acid (5-ALA) and its metabolite protoporphyrin IX in fluorescence-guided tumor resection to identify residual tumor or a vague tumor boundary is already established; the use of 5-ALA PDD is reported to improve the tumor resection rate and patient prognosis (8–10). In PDT for brain tumors, a tumor-selective photosensitizer is administered before surgery, and an excitation light of the appropriate power and wavelength is applied to the tumor or its removal cavity. When illuminated with such light, the photosensitizer is activated and exhibits a tumoricidal effect by producing singlet oxygen with oxidation properties only in the tumor cells, which suppresses the growth of residual infiltrative tumor cells (11, 12). Talaporfin sodium (TS: mono-l-aspartyl chlorine e6, or NPe6) is a second-generation photosensitizer used in intraoperative PDT for malignant brain tumors. PDT with TS was shown to have good clinical safety and efficacy and excellent outcomes, which led to its regulatory approval in Japan (13–15); notably, this method has recently been described as a promising tool for the treatment of malignant brain tumors (11). Because TS and 5-ALA are both porphyrin derivatives (cyclic tetrapyrroles) (16), they both have an absorption peak at 400 nm. Hence, TS not only produces singlet oxygen but also emits fluorescence upon absorbing the appropriate excitation light. In fact, there are several *in vitro* and *in vivo* studies describing the fluorescence from TS (16–19). Thus, we reasoned that TS has the potential to serve as a PDD photosensitizer. If so, PDD with TS (TS-PDD) could be highly beneficial for achieving maximal tumor resection by detecting residual tumor, followed by applying PDT with TS, given that 5-ALA and TS are not used in combination due to the potential for exacerbated side effects (hyper-photosensitivity).

Therefore, the objective of this study was to demonstrate the feasibility of PDD using the fluorescence from TS, which is used for intraoperative PDT, for malignant gliomas. To the best of our knowledge, this is the first clinical study showing that TS can simultaneously serve as a photosensitizer for intraoperative PDD and for the intraoperative PDT of malignant glioma in humans.

## Materials and Methods

### Selection of Patients

Patients were consecutively recruited to this prospective, observational study from May 2016 to April 2017 at the Department of Neurosurgery, Tokyo Women’s Medical University. Inclusion criteria were aged 20 years or older, with a preoperative diagnosis of newly diagnosed or recurrent primary malignant (WHO histopathological grade III or IV) glioma based on neuroimaging, and eligible for surgical removal of the tumor and PDT with TS. Exclusion criteria included a history of photosensitivity or porphyria (contraindication for TS), and biopsy cases. All surgical procedures and data collection were performed at the Tokyo Women’s Medical University Hospital. The research protocol was conducted according to the principles expressed in the Declaration of Helsinki and approved by the local ethics committees of Tokyo Women’s Medical University and Tokyo Medical and Dental University. Each patient was given a written document and provided signed informed consent.

### Surgical Procedures and Tissue Sampling

The enrolled patients received a single intravenous administration of TS (Laserphyrin, Meiji Seika Pharma Co., Ltd., Tokyo, Japan) at a dose of 40 mg/m^2^ body surface area, 22–26 h before elective craniotomy. Tumor removal was conducted using established procedures, without TS-PDD. Tissue samples were obtained from the contrast-enhanced (CE) region for intraoperative rapid pathological diagnosis (IRD) and measurement of fluorescence intensity. After removal of the CE region, we took several tissue samples from the removal cavity wall. These were non-contrast-enhanced (NCE) tissues at the boundary between the tumor and the surrounding normal tissue, as confirmed by a neuronavigation system with 0.4 T intraoperative MRI (iMRI, APERTO Lucent, Hitachi Ltd.). The first iMRI was performed after craniotomy and CSF aspiration to avoid brainshift. After resection of the tumor, a second scan was acquired. If necessary, additional scans were performed at intervals determined by the surgeon. Neuronavigation (Cranial Navigation Application, Brainlab AG) was performed using intraoperatively acquired images (slice thickness: 1 mm) throughout the surgery. The CE region was confirmed by a T1-weighted image with contrast (Meglumine Gadopentetate, 74.28 mg/kg), while the NCE region was confirmed by a T2-weighted image. Every sample was taken using the same forceps, to obtain equal sizes (3–5 mm in diameter). If a sample was resected *en bloc*, a smaller portion of the tissue was taken for the following analyses. For each sample, the fluorescence intensity was measured *ex vivo* (as described in the next subsection), and rapid pathological diagnosis was performed. If the iMRI showed residual CE region, or the IRD revealed significant tumor cells, we performed additional removal. After tumor resection, a PDT laser (PD laser BT, Meiji Seika Pharma Co., Ltd., Tokyo, Japan) was applied to the tumor removal cavity, in accordance with regulations (13–15).

### Fluorescence Measurement

**Figure 1:**
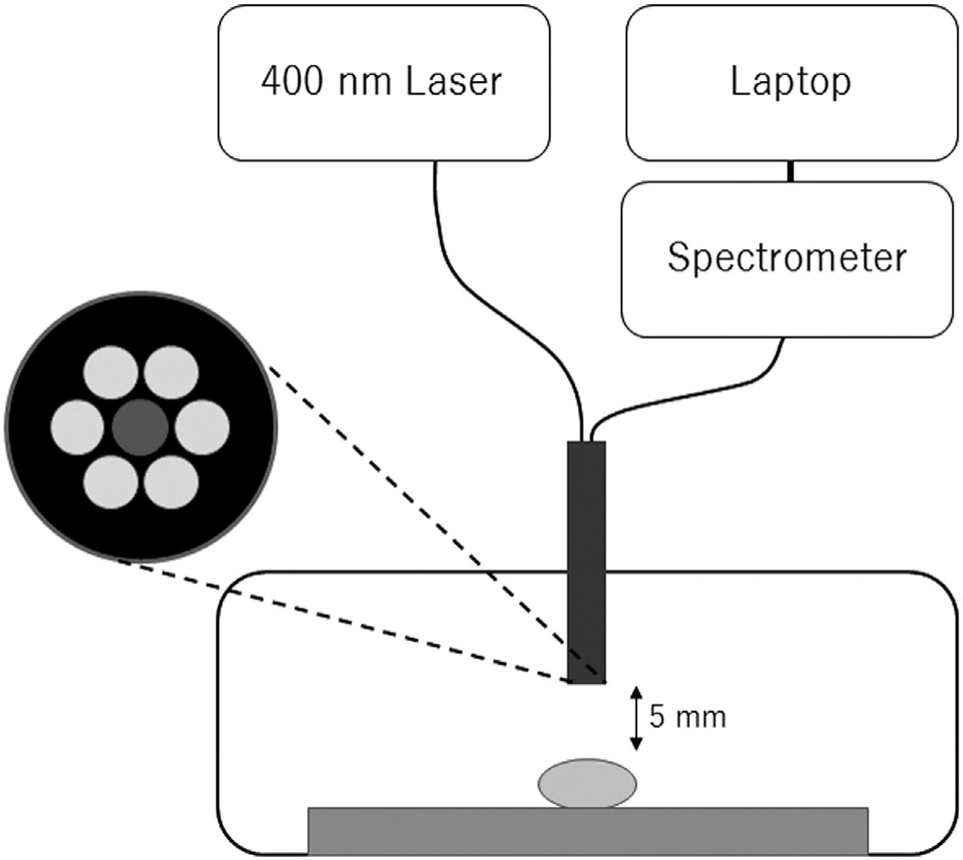
Configuration of the fluorescence detection system. A semiconductor laser unit with a wavelength of 400 nm was used as the excitation light source. A compact spectrometer and laptop were used for the fluorescence measurement. These components were connected to a custom-made probe consisting of an optical fiber bundle (each 0.4 mm in diameter). The fiber at the center was used to collect the fluorescence from the sample, and the others were used to deliver the excitation light. The tumor sample was placed on a glass slide in a 3D-printed box, where the distance between the tumor sample and probe was fixed in a dark environment. The measurement was performed during surgery.

Quantitative spectroscopic analysis of the fluorescence from brain tumor samples has been reported using 5-ALA (20, 21). Figure 1 shows a diagram of the fluorescence detection system used in this study. A semiconductor laser unit with a wavelength of 400 nm (LDS1005BL, Precise Gauges Co., Ltd., Shizuoka, Japan) was used as an excitation light source, because TS has an absorption peak at 400 nm (16). A custom-made probe consisting of seven coaxial optical fibers (numerical aperture = 0.22, Ocean optics, FL, USA) was used to deliver the excitation light and to collect the fluorescence. The fluorescence spectrum (wavelength 390–760 nm) was measured by a compact spectrometer with 2048 cooled CCD arrays and 16-bit digitizers built in (BTC112E and BWSpec 3.26, B&W Tek, DE, USA) and a laptop connected *via* USB. Two tips on one end of the probe were connected to the laser unit and spectrometer, respectively. A tip on the other end was fixed to a measurement box. This box was 3D-printed with resin material to ensure a dark environment and to fix the relative positions of the probe and sample. The excitation light power at the tip of the probe was 15 mW, which was measured by a PowerMax-USB sensor (Coherent Inc., CA, USA; data not shown). The distance between the probe and the sample was approximately 5 mm, resulting in a circular measurement area 2.6 mm in diameter. The fluorescence intensity was calculated as the height of the peak corresponding to TS minus the baseline background at 600 nm. The exposure time was set to 1,000 ms for all samples. This measurement was performed in the operating room, soon after the samples were obtained. Note that this feasibility study was done on *ex vivo* tissue, and not in the surgical field *in situ*. This is because this study was a “first in human” investigation of TS-derived fluorescence in a clinical setting.

### Histopathological Analysis

All tumor samples were subjected to histopathological analysis based on hematoxylin and eosin (H&E) staining, by an experienced neuropathologist who was blinded to the fluorescence data. Tumor cellularity was assessed according to the cell density and morphological appearance. Accordingly, each sample was semi-quantitatively classified into the following four categories (22): class 0, no tumor cells with normal brain appearance; class 1, minor infiltration of tumor cells with rare reactive astrocytes; class 2, mild-to-moderate presence of tumor cells; and class 3, dense and abundant tumor cells with abnormal tissue. The IDH-1 mutation status and Mib-1 index were immunohistochemically assessed by R132H and MIB-1 antibodies, respectively. The tumor grade was determined according to the WHO 2016 guidelines.

### Statistical Analyses

Fluorescence intensity, as a continuous variable, was compared by non-parametric analysis. The Mann–Whitney *U* test and Steel–Dwass test were used to compare two groups and multiple groups, respectively. Trend analysis by the Jonckheere–Terpstra test was also used. Receiver operating characteristic (ROC) analysis was performed to assess the diagnostic performance of the fluorescence intensity for identifying the presence of pathological cells. All *P*-values were two sided, and *P*-values of 0.05 or less were considered statistically significant. All statistical analyses were conducted using EZR version 1.32 (Saitama Medical Center, Jichi Medical University, Saitama, Japan) (23), which is a graphical user interface for R (The R Foundation for Statistical Computing, Vienna, Austria).

## Results

### Study Characteristics

Table 1 summarizes the patient characteristics. Nineteen surgical cases were initially enrolled in this study. Two of them were excluded because the final pathology revealed pilocytic astrocytoma (WHO grade I) in one case and necrosis in the other. Three of the remaining patients underwent tumor removal and PDT twice. Ultimately, 14 patients (3 of whom had repeated surgery), 17 surgical cases (11 males and 6 females), with a median age of 47 (range 36–72 years), were included in the analysis. GBM was the most frequent diagnosis, accounting for 82% of the cases. Seven of the tumors (41%) were newly diagnosed and 10 (59%) were recurrent. A total of 86 samples (41 from CE regions, 45 from NCE regions) were available for fluorescence measurement (5.1 samples per case). Gross total resection (>95% removal of CE tumor) was achieved in all cases; therefore, partial removal and biopsy-only cases were not included in this study.

**Table 1:** Characteristics of 14 patients (17 operations).

Case no.^a^	Age range (years)	Tumor status	Localization	Histology (WHO grade)	IDH-1	Mib-1 (%)
1	40–44	Rec	Right temporal	AE (III)	WT	28.4
2	45–49	New	Right parietal	GBM (IV)	WT	24.5
3	70–74	New	Right frontal	GBM (IV)	WT	14.9
4	50–54	New	Left parietal	GBM (IV)	WT	16.6
5	45–49	New	Left parietal	GBM (IV)	WT	25.3
6	40–44	Rec	Left frontal	GBM (IV)	MT	26.8
7	65–69	Rec	Left temporo-occipital	GBM (IV)	WT	15.1
8	70–74	New	Right temporal	GBM (IV)	WT	45.8
9	35–39	Rec	Right frontal	GBM (IV)	WT	33.3
10	60–64	Rec	Left temporo-parietal	GBM (IV)	WT	16.2
11	40–44	Rec	Right temporal	GBM (IV)	WT	17.2
12	45–49	Rec	Left parietal	GBM (IV)	WT	NA
13	35–39	Rec	Left parietal	AE (III)	WT	24.8
14	40–44	Rec	Right temporal	AE (III)	WT	16.1
15	45–49	New	Left temporal	GBM (IV)	WT	6.7
16	40–44	Rec	Left frontal	GBM (IV)	MT	16.7
17	55–59	New	Left temporal	GBM (IV)	WT	35.2

*^a^The patients were the same in cases 1 and 14; 5 and 12; and 6 and 16*.

*AE, anaplastic ependymoma; F, female; GBM, glioblastoma multiforme; M, male; MT, mutant; New, newly diagnosed; Rec, recurrent; WT, wild type*.

### CE versus NCE Regions

**Figure 2:**
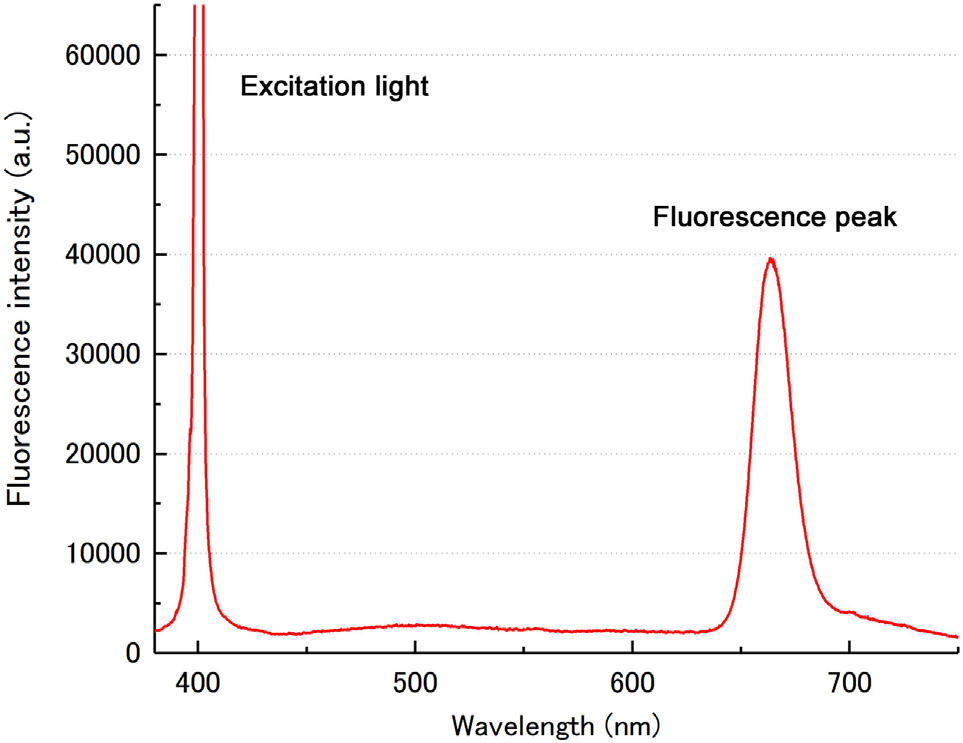
An example of a fluorescence spectrum from the CE region. A single sharp peak at 664 nm indicated the fluorescence from TS. Another peak corresponding to the 400-nm excitation light was easily distinguished from the TS peak. a.u., arbitrary units; CE, contrast-enhanced; TS, talaporfin sodium.

**Figure 3:**
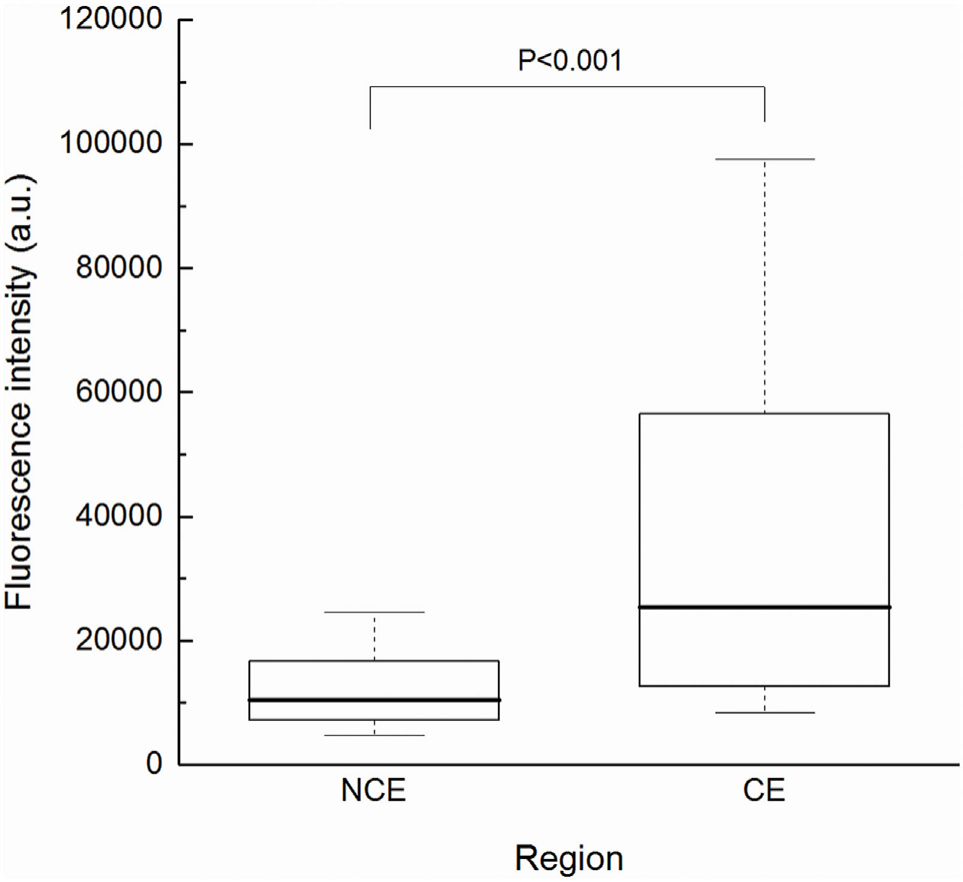
Fluorescence intensity according to tissue region. The CE group (*n* = 41) showed significantly higher intensity than the NCE group (*n* = 45) by Mann–Whitney *U* test (*P* < 0.001). Each box indicated the interquartile range, and the median was shown with a bold line. The ends of the whiskers represented the 10th and the 90th percentile. a.u., arbitrary units; CE, contrast-enhanced; NCE, non-contrast-enhanced.

**Figure 4:**
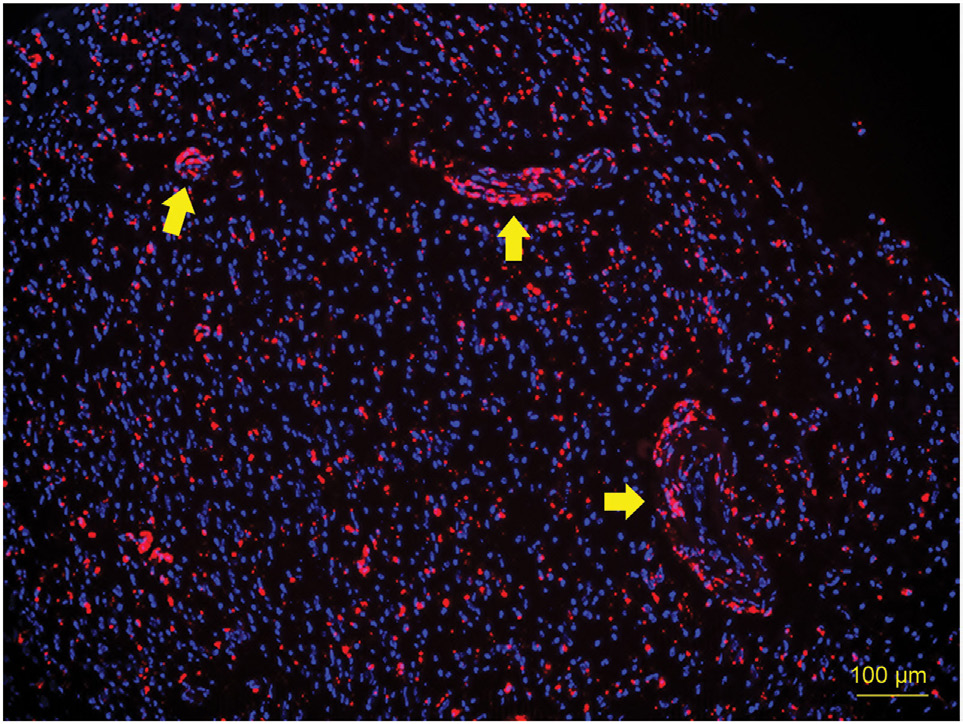
Fluorescence microscopy image of a 10-µm cryosection of the CE region. Tumor cell nuclei were stained with DAPI. Red fluorescence above 600 nm was observed in the cytoplasm of each tumor cell (excitation wavelength 390–410 nm), indicating that TS was incorporated into the tumor cells. Fluorescence was notably distinguishable along vascular endothelium (arrows). CE, contrast-enhanced; TS, talaporfin sodium.

**Figure 5:**
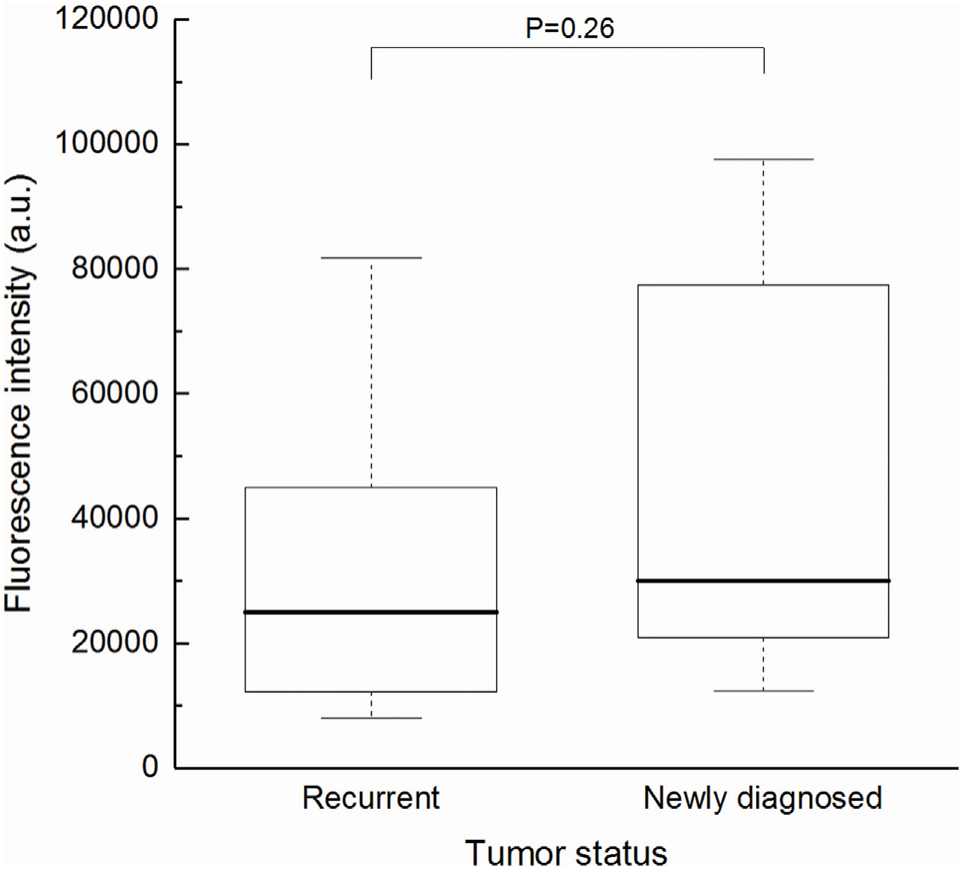
Fluorescence intensity of samples from the CE region of newly diagnosed (*n* = 11) and recurrent (*n* = 30) cases. There was no significant difference between the two groups (Mann–Whitney *U* test, *P* = 0.26). Each box indicated the interquartile range, and the median was shown with a bold line. The ends of the whiskers represented the 10th and the 90th percentile. CE, contrast-enhanced.

First, to determine if TS-PDD for glioblastoma was feasible, we analyzed the fluorescence intensity of the CE regions. An example of a fluorescence spectrum from a CE region is shown (Figure 2). TS had a single peak at 664 nm that was easily distinguished from the excitation light at 400 nm. As expected, samples from the CE regions showed significantly higher fluorescence intensity than those from the surrounding NCE regions (Figure 3, *P* < 0.001). In addition, to confirm that TS was incorporated into the tumor cells, frozen specimens of the CE region were sliced at 10 µm, and the tumor cell nuclei were stained with DAPI. Fluorescence microscopy (BZ-X700, Keyence, Osaka, Japan) revealed red fluorescence in the cytoplasm of each tumor cell. The fluorescence was notably distinguishable along vascular endothelium (Figure 4). The fluorescence intensity between newly diagnosed and recurrent cases was not significantly different (Figure 5, *P* = 0.26). Only samples from the CE region were included in this comparison, because we were focused on the fluorescence emission from the tumor.

### Histopathological Analysis

**Figure 6:**
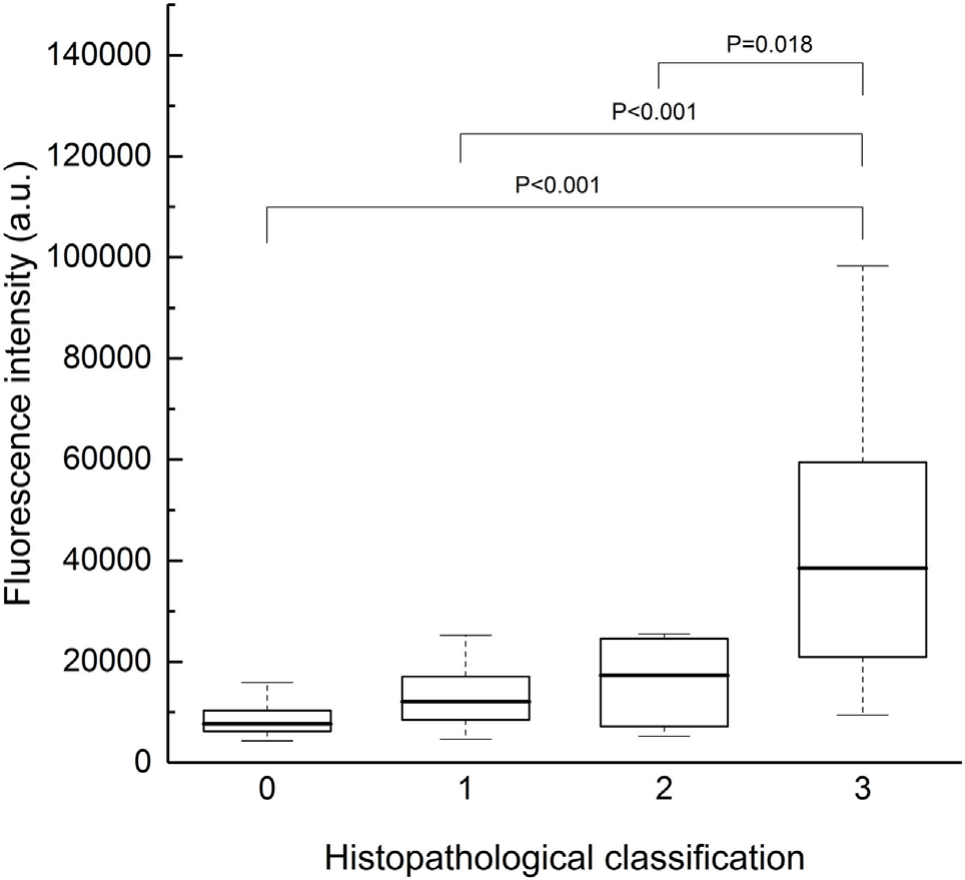
Fluorescence intensity of all samples, plotted according to histopathological classification (0–3). Class 3 samples exhibited markedly strong fluorescence, and a significant increasing trend with classification was observed (Jonckheere–Terpstra test, *P* < 0.001). The Steel–Dwass test revealed statistically significant differences between class 0 and 3 (*P* < 0.001), class 1 and 3 (*P* < 0.001), and class 2 and 3 (*P* = 0.018); the other comparisons were not significant. Each box indicated the interquartile range, and the median was shown with a bold line. The ends of the whiskers represented the 10th and the 90th percentile. a.u., arbitrary units; CE, contrast-enhanced; NCE, non-contrast-enhanced.

**Figure 7:**
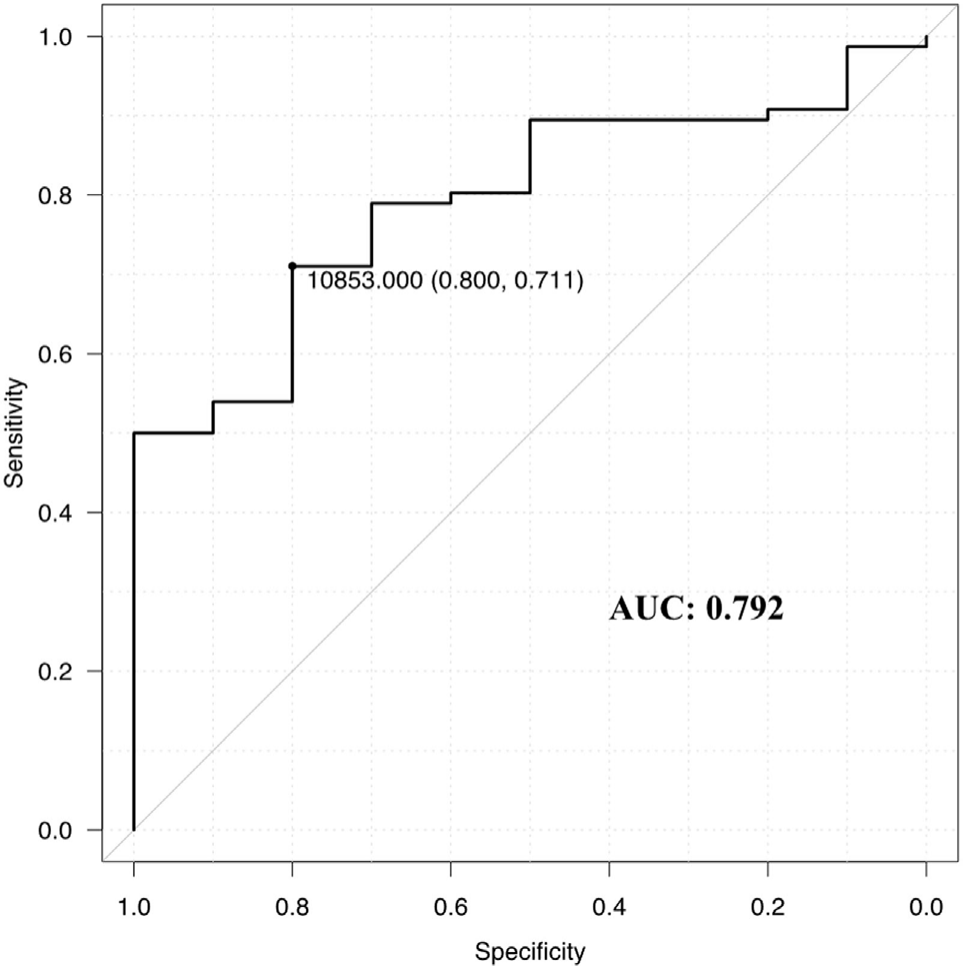
Receiver operating characteristics curve for determining the efficacy of the spectrometry for talaporfin sodium. The specificity and sensitivity were 0.80 and 0.71, respectively, when the fluorescence intensity threshold was set at 10,853 (arbitrary units) for discriminating samples of histopathological class 0, defined as tumor-negative. Area under the curve (AUC) = 0.792, 95% confidence interval: 0.672–0.912.

Next, to assess the intraoperative rapid diagnosis of tumor margins, we compared the fluorescence intensity of all samples according to their histopathological classification (Figure 6). The fluorescence intensity was markedly high in class 3, and an increasing trend of fluorescence intensity with classification was confirmed by the Jonckheere–Terpstra test (*P* < 0.001). Moreover, the Steel–Dwass test revealed statistically significant differences between class 0 and 3 (*P* < 0.001), class 1 and 3 (*P* < 0.001), and class 2 and 3 (*P* = 0.018). A ROC curve was then drawn using fluorescence intensity as a diagnostic value (Figure 7). In diagnosing the resection stump, we assumed that histopathological class 0 indicated tumor-negative. Thus, the threshold was set between histopathological class 0 and 1 for discriminating the presence of tumor cells. The area under the curve was 0.792 (95% confidence interval: 0.672–0.912), and the specificity and sensitivity were 0.80 and 0.71, respectively, using 10,853 (arbitrary units) as the fluorescence intensity threshold.

### Representative Case

**Figure 8:**
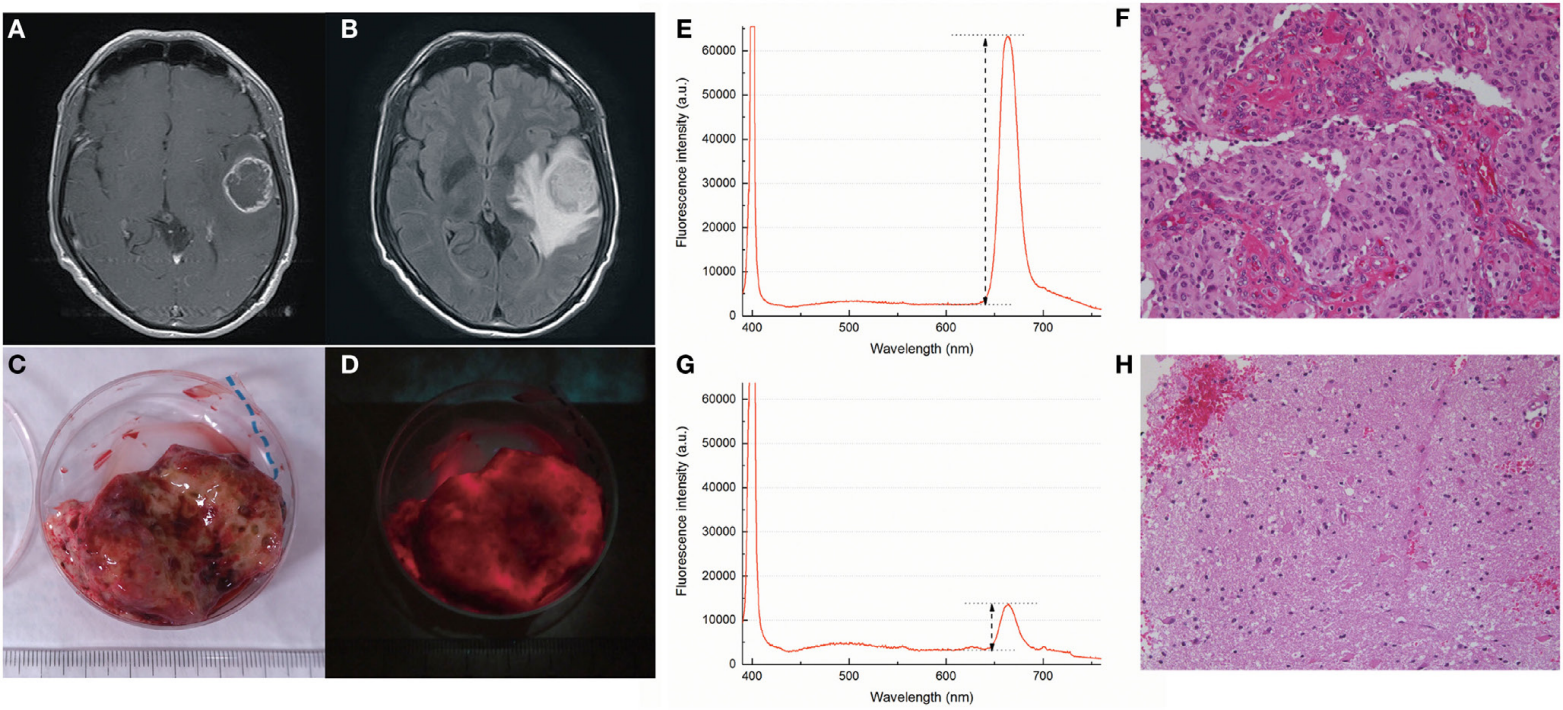
Illustrative case. A 47-year-old male with newly diagnosed glioblastoma multiforme (GBM) in the left temporal lobe. **(A)** Preoperative T1-weighted MR image with contrast showed a tumor in the left temporal lobe with ring-like enhancement (CE region). **(B)** Preoperative FLAIR image revealed severe peripheral edema (NCE region) surrounding the tumor mass. **(C,D)** Intraoperative images of the resected tumor under white light **(C)** and blue laser **(D)** observed through an optical filter (460 nm long pass). Bright red fluorescence was confirmed, particularly around the rim area that corresponded to the CE region. **(E,F)** Spectrum and hematoxylin and eosin stained section (original magnification ×200) obtained from the CE region. The sharp peak at 400 nm was from the excitation laser and the wider peak at 664 nm was from the TS fluorescence **(E)**. Fluorescence intensity was calculated as the peak height (arrows) minus the baseline value at 600 nm (dotted line at the bottom). Abundant tumor cell invasion and endothelial proliferation was seen **(F)**. **(G,H)** The same data obtained from the NCE region. Compared to the CE region, a lower fluorescence intensity **(G)** and reduced cellularity with normal brain tissue **(H)** were observed. a.u., arbitrary units; CE, contrast-enhanced; NCE, non-contrast-enhanced; TS, talaporfin sodium.

Figure 8 shows an illustrative case. A 47-year-old male with no past medical history presented with headache. MRI showed a tumor with low intensity on T1-weighted images, mixed high intensity on T2-weighted images, ring-like enhancement by gadorinium, and severe peripheral edema around the tumor on the FLAIR image (Figures 8A,B). Gross total removal of the CE region was achieved under awake craniotomy with neuronavigation. The resected specimen exhibited bright red fluorescence under blue laser, whereas the necrotic lesion at the center of the sample showed almost no fluorescence (Figures 8C,D). The fluorescence was strong enough to see easily by the naked eye. The fluorescence spectra and corresponding histopathological images are presented in Figures 8E–H. In samples from the CE region, two clear peaks were present: a sharp peak at 400 nm from the excitation laser, and a wider peak at 664 nm from the TS fluorescence. Microscopically, the H&E-stained sample corresponding to the strongly fluorescent region showed abundant tumor cells with nuclear atypia and endothelial proliferation (Figure 8F, original magnification ×200). In another sample obtained from the surrounding NCE region, which represented the high-intensity area on the FLAIR image, a small peak was still present at 664 nm, but its intensity was relatively low (Figure 8G). Under microscopic observation, this sample showed a small number of infiltrating tumor cells on a background of normal brain tissue (Figure 8H, original magnification ×200).

## Discussion

Several *in vivo* and *in vitro* studies have described the fluorescence from cells or tissues treated with TS (16–19), and a small case series examined the possibility of PDD with TS (in Japanese) (24). However, this is the first clinical study to demonstrate the feasibility and diagnostic value of TS-induced red fluorescence acquired intraoperatively in patients with malignant glioma, using blue excitation light. In this study, we measured the fluorescence from various tumor samples obtained from patients who underwent PDT with TS. We used the histopathological assessment by a neuropathologist as a reference for tissue malignancy. The results showed that, as expected, tumor samples from the CE regions, which were often rich in vascular structures, exhibited significantly stronger fluorescence than those from the surrounding NCE regions (Figure 3). This technique was applicable for both newly diagnosed and recurrent cases (Figure 4). In addition, samples with higher tumor cellularity were associated with stronger fluorescence (Figure 6). These findings were consistent with the previously reported tumor selectivity of TS (17). ROC analysis revealed that the tumor-negative tissue was well discriminated according to the fluorescence intensity (specificity = 0.80; sensitivity = 0.71). These results indicated that our new technique might enable accurate PDD of the residual CE lesion that must be completely removed from the surrounding NCE tissues. Moreover, due to their similar optical properties, available tools for 5-ALA PDD could be applied to TS-PDD. In fact, TS’s red fluorescence, which is similar to 5-ALA-induced fluorescence, was easily visible in the tumor (Figure 8D).

In general, when a photosensitizer absorbs the appropriate light energy, it is excited and reaches a state called the excited singlet state. As this state is unstable, the photosensitizer returns to its basal state by releasing energy in various ways, one of which is the emission of fluorescence. Another way is the transition to another state called the excited triplet state, which has a tumoricidal effect by producing reactive oxygen species (11, 14, 25). These are the two major photodynamic properties exploited in brain tumor surgical techniques, suggesting that PDD and PDT could be performed simultaneously. However, administering two different photosensitizers such as 5-ALA and TS could lead to hyper-photosensitivity-related complications, and the safety of such dual administration has not been established. Therefore, the use of TS for both PDD during tumor resection and for PDT after maximal tumor removal has high-clinical potential. In the approved clinical protocol for PDT with TS in Japan, a red laser is used because red light penetrates deeper into brain tissues than the blue light used in 5-ALA PDD, and it avoids absorption by hemoglobin (17). When illuminated at 664 nm, however, TS emits a similar red fluorescence (672 nm) (14) that is indistinguishable by the human eye and requires a specialized filter for separation. Thus, we chose a blue laser (400 nm) as the excitation light for TS-PDD that is also used for 5-ALA PDD, because the two photosensitizers have an absorption peak in common (called the Soret band) at around 400 nm (16, 17, 25). This technique would be practicable, because the equipment for 5-ALA PDD is commercially available.

We note an important difference in the distribution of photosensitizers in human tissues. Unlike 5-ALA, TS is administered *via* intravenous injection and involves no metabolic pathway. The basic principle of its tumor selectivity is considered not to be metabolism, but rather its affinity to albumin and its possible association with transporters such as ABCG2, resulting in its accumulation in lysosomes (17, 26). Hence, blood and blood vessels themselves may exhibit fluorescence. In fact, we demonstrated that the fluorescence in tumors was prominent along vascular endothelium (Figure 4). In addition, some of the blood around the tumor samples showed fluorescence (Figure 8D). Considering the histopathological nature of GBM, the strong fluorescence in samples with the highest tumor cellularity could be associated with microvascular proliferation in the malignant glioma tissue (13).

Our technique, TS-PDD, should help surgeons identify the residual tumor intraoperatively, because the CE region and tumor tissue with higher cellularity emitted bright fluorescence. Using the methodology that has been approved in Japan, PDT is effective on the irradiated surface only to a depth of approximately 5 mm (13, 15). Therefore, maximal resection (cytoreduction) of the tumor is required before performing PDT (13). Hence, PDD using the same photosensitizer would be a useful option to include. The PDD technique is essential for institutions that are not equipped with intraoperative MRI, which is used to evaluate residual brain tumor definitively. PDD has the advantage of being an easy-to-introduce, real-time measurement. To apply this technique in clinical practice, the standard approach and equipment used for fluorescence-guided resection with 5-ALA could be used. Illumination of the tumor tissue with blue excitation light would generate red fluorescence, like 5-ALA-induced fluorescence. Although the tumoricidal and photobleaching effects of this blue light have not been investigated in detail, we believe they are limited, because blue light has a low penetration depth. To increase penetration, longer wavelengths at around 650 nm are frequently used in PDT (11). In addition, in the PDD application, the illumination time is shorter than the duration of TS treatment with a red laser (3 min each spot). In any case, the residual CE tumor tissue can be identified with our simple method, although recognition of the precise resection boundary will require further study. In addition, caution should be exercised in the presence of blood vessels. Large vascular structures may exhibit fluorescence even in the absence of tumor. Thus, as in performing PDT with TS, large vessels must be kept shielded from the treatment laser to avoid embolism. In fact, microvasculature damage and its subsequent effect on the vascular system surrounding the tumor was identified as a basis for TS’s antitumor activity when it was first introduced in Japan for treating early stage lung cancer (27). The fluorescence emission from blood vessels might explain why the area under the ROC curve (0.792) for TS in our study was a bit smaller than that reported for 5-ALA (0.88) (28).

There are several limitations to acknowledge in this study. First, although the fluorescence was measured quantitatively, variations in the tumor sample size could have caused deviations in the true fluorescence intensity, by affecting the distance to the detection probe. Second, there was lack of objective assessment in determining the tumor cellularity, even by the experienced neuropathologist. Nevertheless, this approach has been used and reported elsewhere (22). Another minor limitation was the small number of grade III tumors. Most of the cases were GBM, and there were no anaplastic astrocytoma or anaplastic oligodendroglioma cases included in this study. Since TS PDT is applicable for grade III and IV (high-grade) gliomas, the application of our method for these tumors might require further investigation.

Despite these limitations, this study indicated that TS is feasible as a PDD photosensitizer in patients with malignant glioma, particularly GBM, for the first time in humans on *ex vivo* tissue. Ultimately, we believe that TS-PDD would help maximize the extent of tumor resection, leading to better effectiveness of PDT and better outcomes for patients. Further investigation in a prospective study using TS-PDD *in situ* is needed.

## Conclusion

This study demonstrated that red fluorescence arose in tumor samples from patients with malignant glioma who were undergoing PDT with TS. Tumor samples from the CE region exhibited significantly stronger fluorescence than those of NCE regions. Histopathological analysis confirmed an increasing trend of fluorescence intensity with tumor cellularity. The involvement of microvascular structures could help explain the strong fluorescence from pathologically malignant tissues. These results suggested that TS might enable intraoperative PDD and PDT to be performed simultaneously for patients with malignant glioma. In addition, due to their similar optical properties, the tools for 5-ALA PDD could be used for TS-PDD.

## Ethics Statement

The research protocol was conducted according to the principles expressed in the Declaration of Helsinki and approved by the institutional ethics committees of Tokyo Women’s Medical University (3842-R) and Tokyo Medical and Dental University (M2015-580). Each patient was given a written document and provided signed informed consent.

## Author Contributions

TK and TMae supervised the project. KS, YM, and MN developed the concept and the design of the study. KS and KM designed and made the experimental setup for data acquisition. MN, TMar, TY, YF, and YM carried out patient care, surgery, and sample acquisition. KS performed data collection. TKo made significant contributions to histopathological analysis. KS, MN, TKo, TMar, TY, YF, KM, and YM analyzed and interpreted the data. KS drafted the manuscript. MN, TKo, and YM critically revised the manuscript. All authors have approved the final manuscript.

## Conflict of Interest Statement

The authors declare that the research was conducted in the absence of any commercial or financial relationships that could be construed as a potential conflict of interest.
